# Surgical management of foregut duplication cyst existing with a congenital diaphragmatic hernia: A case report

**DOI:** 10.1016/j.ijscr.2024.110369

**Published:** 2024-09-30

**Authors:** Katherine Bruckner, Maho Kurashima, Christopher Blewett, Shin Miyata, Richard Herman

**Affiliations:** Saint Louis University School of Medicine and SSM Health Cardinal Glennon Children's Hospital Department of Pediatric Surgery, 1465 S Grand Blvd, STL, MO 63104, United States of America

**Keywords:** Congenital diaphragmatic hernia, Foregut duplication cyst, Case report

## Abstract

**Introduction:**

There have been two cases of congenital diaphragmatic hernia associated with a gastroesophageal duplication cyst documented in the literature, both presented symptomatically in the newborn period. This case is unique given the patient presented in adolescence asymptomatically.

**Presentation of case:**

We present a case of a 15-year-old female who initially presented with lower abdominal pain. A CT chest and abdomen was concerning for a moderate to large hiatal vs diaphragmatic hernia and an adjacent 4.3 cm lower mediastinal cyst. Though asymptomatic, the patient underwent diagnostic thoracoscopy with cyst excision. On post operative day one, the patient endorsed severe uncontrolled abdominal pain with multiple bouts of non-bilious emesis. Chest X-ray and upper GI were positive for herniation of the stomach through the diaphragm. The patient was taken back to the operating room emergently for diagnostic thoracoscopy where the stomach was found herniated through a diaphragmatic defect with no evidence of necrosis. The stomach was reduced into the abdominal cavity. The patient tolerated this procedure well, recovered appropriately and was seen two-weeks post-operatively fully recovered.

**Discussion:**

Better visualization and thorough tactile check of the diaphragmatic defect during the initial operation would increase the chance of detecting a defect in the diaphragm immediately and avoid subsequent herniation of abdominal contents.

**Conclusion:**

When Foregut duplication cysts exist near the diaphragm it is critical to get a thorough view of the anatomy and ensure no diaphragmatic defect exits.

## Highlights

• Foregut duplication cysts (FDC) may present asymptomatically.
• Surgical management FDC may be indicated to avoid complications.
• FDC near the diaphragm requires a thorough view of the anatomy and assessment of diaphragmatic integrity to close defects.

## 1 Introduction

Duplication cysts and congenital diaphragmatic hernias are rare as duplication cysts are present in approximately 1 in 4500 births and congenital diaphragmatic hernias are present in approximately 1 in 3600 births [1,2]. It is especially atypical for duplication cysts and diaphragmatic hernias to exist simultaneously. There have been two cases of congenital diaphragmatic hernia associated with a gastroesophageal duplication cyst documented in the literature. We present a case of a 15-year-old female who initially complained of lower abdominal pain and was subsequently diagnosed with a diaphragmatic hernia and foregut duplication cyst.

## 2 Case report

**Fig. 1:**
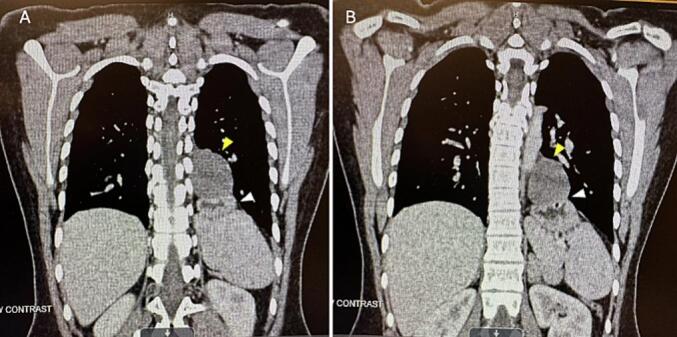
Chest computed tomography with contrast preoperative. Moderate to large left diaphragmatic/hiatal hernia (white arrow). Adjacent 4.3 cm lower mediastinal cyst (yellow arrow) most likely representing benign pathology such as bronchogenic or pericardial cyst. (For interpretation of the references to colour in this figure legend, the reader is referred to the web version of this article.)

A previously healthy 15-year-old female initially presented to the emergency department endorsing lower abdominal pain and was diagnosed with mesenteric adenitis. Upon workup CT suggested a large hiatal hernia (type I) and 4.6 cm posterior mediastinal cyst. She presented for surgical evaluation one week later at which time her abdominal pain had improved, and she denied any symptoms associated with the hiatal hernia such as reflux, issues tolerating diet, weight loss, or emesis. Subsequent CT chest and abdomen (Fig. 1) was again concerning for a moderate to large hiatal vs diaphragmatic hernia and adjacent 4.3 cm lower mediastinal cyst thought to be an esophageal duplication, bronchogenic or pericardial cyst. Given the size and location of the cyst, cyst removal was recommended. Because the patient was asymptomatic and the CT scan was concerning for a large hiatal hernia, she underwent surveillance EGD to assess for the presence of the hiatal hernia and for any possible mucosal changes. The EGD did not show any evidence of a hiatal hernia, and all biopsies were normal. As such, preoperatively it was felt the read of the CT was incorrect and the hiatal hernia seen on the CT scan was truly the duplication cyst.

The patient underwent diagnostic thoracoscopy with cyst excision. Four ports were placed in the following locations: tip of the scapula, mid axillary line at the 4th intercostal space, middle axillary line at the 6th intercostal space, and the mid-axillary line at the 7th intercostal space. A 5 cm × 5 cm cyst filled with white mucus fluid was found to be in the left chest cavity on the diaphragm separated from the pericardium, aorta, and esophagus. The cyst was adjacent to the hiatus, covered with pleura and mild adhesions (Fig. 3a). The pleura was circumferentially dissected with hook cautery and a vessel sealing system revealing a 2-3 cm stump connecting the cyst to the abdominal cavity and attached to the gastric wall. The pleura stump was skeletonized and resected using a 60 mm laparoscopic stapler. The diaphragm was inspected (Fig. 3b), the staple line retracted and no obvious hiatal hernia or residual diaphragmatic defect was appreciated. Intraoperatively it was confirmed the CT scan was misinterpreted and there was no hiatal hernia. A 20 Fr chest tube was placed in the mid-axillary line at the 7th intercostal space. Pathology revealed a benign cyst lined by epithelium containing respiratory, stratified squamous esophageal, and gastric oxyntic-type epithelial mucosa consistent with a foregut duplication cyst.

**Fig. 2:**
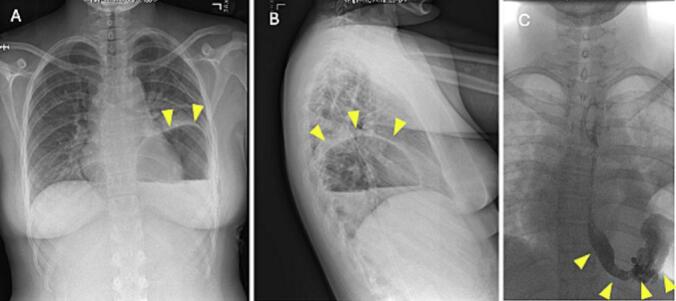
(A) Chest X ray posterior anterior view. (B) Chest X ray lateral view. Increased size of the retrocardiac lucency (arrow), likely representing hiatal hernia (arrow). (C) Upper GI showing loculated area of lucency lateral to the contrast-filled gastric body (arrow) within the left lower hemithorax; this may reflect air-filled stomach, bowel loop, or loculated postoperative pneumothorax.

The patient initially appeared to be recovering well. However, on post operative day one, the patient endorsed severe uncontrolled abdominal pain with multiple bouts of non-bilious emesis. Chest X-ray and upper GI were positive for herniation of the stomach through the diaphragm without leak or clearance of contrast from the stomach (Fig. 2). With suspicion for incarceration of the stomach with volvulus, the patient was taken back to the operating room emergently for diagnostic thoracoscopy.

**Fig. 3:**
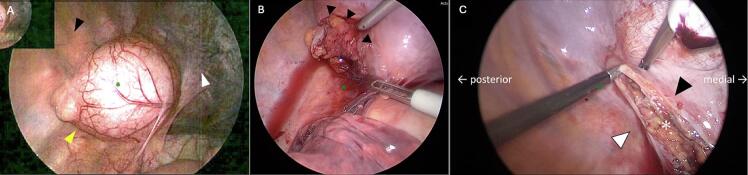
Intraoperative images. (A) Cyst prior to extraction (yellow arrow), pericardium (whit arrow), diaphragm (black arrow). (B) First operation, post cyst extraction, diaphragm (black arrow). (C) Second operation. Anterior leaflet of the diaphragmatic hernia (black arrow), posterior leaflet of the diaphragmatic hernia (white arrow), hernia defect (asterisk). (For interpretation of the references to colour in this figure legend, the reader is referred to the web version of this article.)

In the OR the stomach was found herniated through the diaphragmatic defect with no evidence of necrosis. The stomach was reduced into the abdominal cavity and the defect located at the posterior medial aspect of the left diaphragm separate from the hiatus, the same area where the stump of the cyst was stapled. Again, no evidence of a hiatal hernia was found and no other defects in the diaphragm were appreciated. At this point it was realized the initially suspected hiatal hernia was a type A Bochdalek Congenital Diaphragmatic Hernia, that was missed during the initial operation (Fig. 3c). Primary repair of the defect was performed, and a 20 Fr chest tube was placed in the same location from the day prior. Next, a diagnostic laparoscopy was performed to visualize the stomach which was in the correct anatomical position and healthy with no signs of volvulus. The patient tolerated this procedure well, recovered appropriately and was discharged two days later. She was seen two-weeks post-operatively doing well and fully recovered and was followed up with at one year still doing well.

## 3 Discussion

There have been two documented cases of congenital diaphragmatic hernias associated with a gastroesophageal duplication cyst. Danzer et al. presented a case of an infant with a congenital diaphragmatic hernia discovered prenatally who was later found to have a symptomatic gastroesophageal duplication cyst within the first month of life [3]. Additionally, Secil et al. presented a case of a 4-month baby with an esophageal duplication cyst, diaphragmatic hernia and polysplenia [4]. Our case is unique in that the patient was asymptomatic and the cyst and diaphragmatic hernia were not discovered until she was fifteen years old.

Duplication cysts are most commonly symptomatic within the first two years of life [1]. Depending on where they are found in the alimentary tract they are further divided into foregut, midgut, and hindgut duplications and the embryologic origin of foregut cysts further divides them into esophageal, bronchogenic, and neurenteric [5,6]. When symptomatic foregut duplication cysts may present with a variety of symptoms, depending on the type of duplication. Esophageal duplication cysts may present with dysphagia, vomiting, epigastric pain, and upper gastrointestinal bleeding. Bronchogenic cysts may also present with dysphagia in addition to chest pain, cough, and dyspnea. Finally, gastric duplication cysts may present with abdominal pain, vomiting, abdominal mass and features of gastric outlet obstruction [5]. Occasionally foregut duplication cysts are asymptomatic, as was the case for our patient.

While the etiology of foregut duplication cysts is not fully understood there are multiple theories in the literature including the split notochord, recanalization theory, and partial twinning theory [7]. Duplication cysts are difficult to diagnose and while radiologic imaging has some utility, histologic evaluation is required for definitive diagnosis [3]. Depending on location, duplication cysts may be treated with thoracoscopic excision or an abdominal approach, otherwise open surgery is utilized. Removal is necessary as complications associated with duplication cysts include torsion of the cyst, malignant changes, infection, peptic ulcer, obstruction, hemorrhage, pancreatitis, and fistula formation [8]. Therefore, even though our patient was asymptomatic, surgery was advised given the size of the cyst and possibility of the patient experiencing complications in the future.

In this case, radiologic studies could not definitively differentiate whether this patient had a hiatal hernia or a congenital diaphragmatic hernia. Additionally, this patient's hernia was asymptomatic. It is rare for congenital diaphragmatic hernias to present late given 5–25 % are present after the neonatal period [9]. Late presentations of congenital diaphragmatic hernia typically take on a milder form leading to a more favorable prognosis than neonatal diagnosis [10,11]. However, additional malformations present in later diagnosed congenital diaphragmatic hernias have been documented. In a retrospective study analyzing 20 patients with delayed presentation of congenital diaphragmatic hernia, 80 % of the patients had an associated malformation including malrotation, umbilical hernia, gastroesophageal duplication cyst, etc. [10]. A retrospective study found late-onset congenital diaphragmatic hernias on the left side most presented with gastrointestinal issues and right-sided hernias were associated respiratory symptoms. In the same study, 11 % of cases were asymptomatic [11]. Cruz et al. found patients with congenital diaphragmatic hernias with accompanying space-occupying lesions have a similar prognosis to those with isolated congenital diaphragmatic hernias [12].

This case was especially challenging given the size of the cyst and proximity to the diaphragm and hiatus. Therefore, the risk of damaging the diaphragm during dissection or cyst extraction was increased. The diaphragmatic defect that was repaired in the second operation was the congenital lesion that was interrogated but missed during the first operation, where at the time we felt no repair was needed. No obvious diaphragmatic defect was identified during the initial surgery, even after excision of the duplication cyst stump. In hindsight, the defect could have been obscured by thin pleural or adhesive tissue, and the stomach was kept in the abdomen secondary to the insufflation. Since the staple line was still visible and the stomach was not herniating into the chest, the conclusion was made no diaphragmatic repair was needed. Better visualization of the diaphragmatic defect during the initial operation and thorough tactical check with forceps of the diaphragmatic edge would increase the chance of detecting a defect in the diaphragm immediately and avoid subsequent herniation of abdominal contents. It is vital to closely monitor patients post operatively for herniation and promptly repair the defect to avoid bowel necrosis. As in this case, early intervention leads to favorable results and no long-term consequences.

## 4 Conclusion

Foregut duplication cysts may present asymptomatically, depending on the size and location, surgical management is necessary to avoid further complications. When Foregut duplication cysts exist near the diaphragm it is critical to get a thorough view of the anatomy and perform adequate tactile check with graspers to ensure no diaphragmatic defect exits.

Statements:

1. Informed consent was obtained.
2. The authors have no competing interests to declare.
3. The work has been reported in line with the SCARE criteria [13].

## Consent

Written informed consent was obtained from the patient's parents/legal guardian for publication and any accompanying images. A copy of the written consent is available for review by the Editor-in-Chief of this journal on request.

## Ethical approval

This case report was exempt from ethical approval and was waived by Saint Louis University IRB.

## Funding

There were no sources of funding for this research.

## Author contribution

Katherine Bruckner, B.S.- writing of article

Maho Kurashima, MD, PhD- reviewing article

Christopher Blewett, MD- reviewing article

Shin Miyata, MD- reviewing article

Richard Herman, MD- writing of article

## Guarantor

Richard Herman.

## Conflict of interest statement

The authors have no conflict of interests to declare.
